# Paediatric Contacts of Adult COVID-19 Patients: Clinical Parameters, Risk Factors, and Outcome

**DOI:** 10.1155/2021/2141128

**Published:** 2021-05-24

**Authors:** Ammara Farooq, Taimur Khalil Sheikh, Fibhaa Syed, Tehmina Mustafa

**Affiliations:** ^1^Department of Paediatrics, Federal General Hospital Islamabad, Pakistan; ^2^Department of Paediatrics, Al Nafees Medical College and Hospital, Islamabad, Pakistan; ^3^Department of Medicine, Pakistan Institute of Medical Sciences, Shaheed Zulfiqar Ali Bhutto Medical University, Islamabad, Pakistan; ^4^Centre for International Health, Department of Global Public Health and Primary Care, University of Bergen, Bergen, Norway; ^5^Department of Thoracic Medicine, Haukeland University Hospital, Bergen, Norway

## Abstract

**Background:**

There is insufficient data in Pakistan and in South Asia regarding paediatric COVID-19 demographics and related parameters. The main aim of this study was to assess the paediatric population exposed to SARS-CoV-2 infection, their clinical parameters, risk factors, and outcome.

**Methods:**

This was a descriptive retrospective study conducted at the Pakistan Institute of Medical Sciences and Federal General Hospital Islamabad from 23^rd^ July 2020 to 22^nd^ August 2020. All paediatric contacts (≤13 years) of one hundred adult COVID-19 patients were included. Data of the index cases was taken from the medical records. Paediatric data was collected on the phone using a predesigned proforma.

**Results:**

There were 137 paediatric contacts of 100 adult COVID-19 index cases. The index cases were predominantly males (67%) and belonged to the middle socioeconomic class (89%), and 14% succumbed to the disease. Females had more paediatric contacts. The mean age of contacts was 6.6 years, and the majority (80%) developed no symptoms. Among the symptomatic contacts, fever and cough were the most common symptoms. None of the contacts developed dyspnoea or required hospitalization. Majority of the contacts had been vaccinated with the BCG vaccine. Testing for COVID-19 was done in only 77 (56%) contacts, 25 (32%) by the government team, and 52 (67%) privately. A higher number of symptomatic contacts were positive (15/17 (88%)) as compared to that of the asymptomatic contacts (6/60 (10%)) (*p* = 0.002). Development of symptoms in the contacts was associated with the history of respiratory illnesses, recurrent infections, use of hematinics, a positive COVID-test result, and health professionals being index cases (*p* ≤ 0.01). Parents with higher education and in the health profession and the families of symptomatic contacts reported better compliance with quarantine regulations.

**Conclusion:**

A significant number of children were exposed to adult COVID-19 patients. Most paediatric contacts remained asymptomatic. Children with preexisting medical conditions and with parents in health profession were susceptible to infection.

## 1. Introduction

A new coronavirus was identified in December 2019 in Wuhan, Hubei Province, China, among patients exhibiting respiratory symptoms and a history of exposure to the Huanan seafood market [1, 2]. The World Health Organization (WHO) announced it as a public health emergency of international concern and named the virus as severe acute respiratory syndrome coronavirus 2 (SARS-CoV-2) and the disease as coronavirus disease 2019 (COVID-19) [1]. The age of infected people ranges from 1.5 days to 96 years, and both genders seem to be equally affected. The groups at high risk of severe disease and death are the elderly with underlying conditions such as diabetes, hypertension, and cardiovascular disease. At the peak of the outbreak, children are involved too, including neonates [3, 4].

Most of the children with COVID-19 have low morbidity, and many develop mild symptoms or are asymptomatic, similar to that observed for severe acute respiratory syndrome (SARS) and Middle East respiratory syndrome (MERS). The few severe cases have usually exhibited underlying or coexisting medical conditions [5]. Younger age, underlying pulmonary pathology, and immunocompromising conditions have been associated with serious outcomes with non-SARS-CoV-2 coronavirus infections in children, and the same pattern is reflected in SARS-CoV-2 infection [6].

The debate that children may play a major role in community-based viral transmission is still ongoing. Available data suggest that children may have more upper respiratory tract involvement (including nasopharyngeal carriage) rather than lower respiratory tract involvement [4]. There is also evidence of fecal shedding in the stool for several weeks after diagnosis, leading to concern about fecal-oral transmission of the virus [7]. There has been constant change of views on this point with latest evidence in favor of children not being sources of transmission and thus reopening of schools and daycares can be safely considered [8].

There is insufficient data in Pakistan and in South Asia regarding paediatric COVID-19 demographics and related parameters. The main aim of this study was to assess the paediatric population exposed to SARS-CoV-2 infection, their clinical parameters, risk factors, and outcome.

## 2. Methods

This retrospective descriptive study was conducted at the Pakistan Institute of Medical Sciences and Federal General Hospital Islamabad. Ethical approval was taken from the ethical committee of the mentioned institutes. Our inclusion criteria were all adult patients with COVID-19 admitted at one of the Corona wards at the Pakistan Institute of Medical Sciences, Islamabad, during May 2020-August 2020. A total of 114 patients were admitted during this period. These patients were contacted on telephone for an informed consent. For patients who had expired, consent was obtained from their next of kin. Fourteen patients refused participation and were excluded from the study. Medical records of these 100 patients who gave their consent were obtained from the Pakistan Institute of Medical Sciences.

The paediatric contacts of these patients were identified on the telephone; data was collected on the phone and entered on a predesigned proforma. Paediatric contact was defined as any child from 1 month to 13 years of age exposed from 2 to 14 days to a patient who was positive for COVID-19 and the contact occurred while they were ill as outlined by the National Institute of Health, Pakistan [9]. All children ≤ 13 years of age fulfilling the contact definition were included in the study whereas children who did not meet the contact definition or in whom complete information was lacking were excluded.

Nonprobability random sampling was done. Sample size was estimated based on literature review. The exact sample size was not calculated due to the lack of data available for paediatric patients in Pakistan at the time of study. Contact tracing was defined as tests done by government teams by tracing the contacts of COVID-19 cases, while it was labelled as self-testing when contacts of COVID-19 patients got them investigated on their own from different laboratories without doctor advice.

Data was entered and analyzed using SPSS 26. Descriptive statistics were performed to obtain frequency and percentages of categorical variables. Mean, standard deviation (SD) was reported for continuous variables. Chi-square test and independent *t* test were used for finding statistical significance among different variables. Binary logistic regression analysis was carried out to identify factors associated with the development of symptoms in the contacts. *p* value of <0.05 was considered statistically significant.

## 3. Results

### 3.1. Characteristics of the Index Cases

A total of 100 adult patients diagnosed with COVID-19 were included in our study. All these patients had severe symptoms and hypoxia and required hospital admission. The mean age was 51.4 years (±14.2).  Table 1 shows the characteristics of the index cases. There was predominance of males (67%, *p* = 0.001). A large proportion of patients belonged to the middle socioeconomic class (89%, *p* ≤ 0.001). There was no difference between males and females regarding their disease outcome. Females, on average, had more paediatric contacts as compared to males (*p* = 0.03). While most of the patients recovered form COVID-19, 14% (*n* = 14) succumbed to the disease.

### 3.2. Characteristics of the Paediatric Contacts

**Figure 1:**
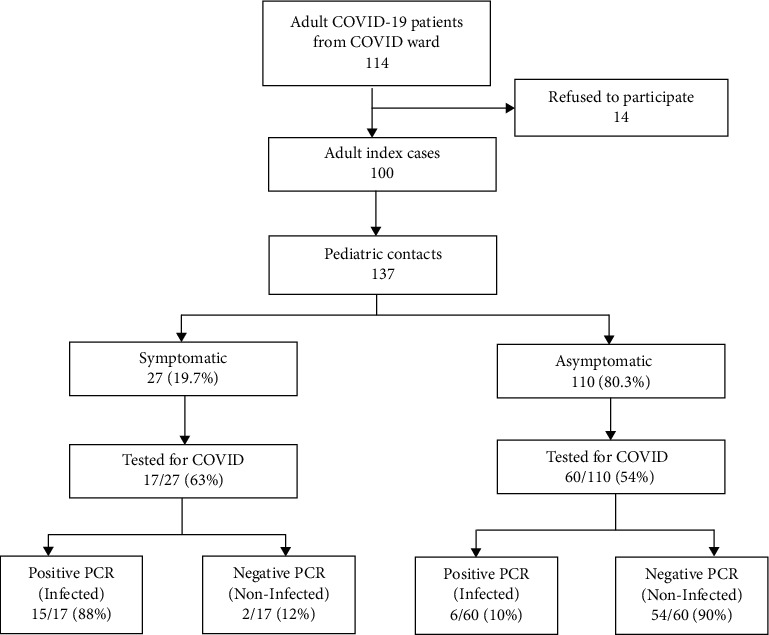
Flow chart of involved contacts and their course of involvement.

Figure 1 shows the flow chart of involved contacts and their course of involvement. Our study identified 137 contacts of ≤13 years of age. Majority of the contacts (80%) developed no symptoms.

Table 2 shows the characteristics of the contacts with and without symptoms. The ages of the contacts ranged from 6 months to 13 years, with a mean age of 6.6 ± 3.6 years. The contacts were exposed to the index cases from 2 to 7 days. There was no difference in the duration of contact between symptomatic and asymptomatic contacts. Among the symptomatic contacts, fever and cough were the most common symptoms. The other less common symptoms were headache, diarrhea, and burning eyes. None of the contacts developed dyspnoea or required any hospitalization. Majority of the contacts had been vaccinated with the BCG vaccine. Very few had also been given the influenza vaccine. Some contacts also suffered from previous respiratory or cardiac illness, such as asthma, ventricular septal defect, and patent ductus arteriosus. Although there was no statistical difference between development of symptoms between contacts with previous cardiac disease and healthy contacts, there was a significant difference in the development of symptoms with previous respiratory disease (*p* = 0.01). Recurrent infections were present in 6 contacts, and it was found that 4 of these were symptomatic, which was statistically significant (*p* = 0.01).

Not all the contacts were tested for COVID-19. Testing was done in 77 (56%) contacts. Out of them, 25 (32%) contacts underwent contact tracing by the government team, of which 5 (20%) turned out to be positive. Another 52 (67%) contacts got testing done on their own from different laboratories, and of these, 16 (30.7%) came out positive. A higher number of symptomatic contacts were positive (15/17 (88%)) as compared to the asymptomatic contacts (6/60 (10%)) (*p* = 0.002). The time of testing was not studied and 44% were not tested, so the actual number of positive cases could not be exactly commented on.

By binary logistic regression analysis, respiratory illnesses, recurrent infections, use of hematinics, and a positive COVID-test result were significantly associated with the development of symptoms among the contacts. The use of hematinics was taken as a proxy for the presence of iron deficiency anemia.

**Figure 2:**
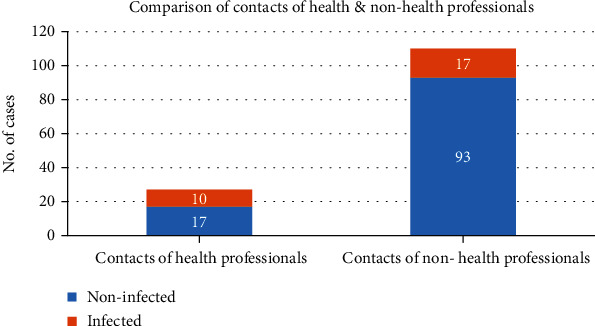
Comparison of contacts of the health and nonhealth professionals. Higher number of contacts of the health professionals developed symptoms (*p* = 0.01) representing health professionals as index cases being a risk factor for transmission of COVID-19 to paediatric contacts.

As mentioned previously, our index cases also included health professionals (nurses or doctors) who were at greater risk of exposure to the disease.  Figure 2 shows the comparison of contacts between the health and nonhealth professionals. Among the contacts, 27 had one or both parents who were health professional, and of these, 10 (37%) developed symptoms. Conversely, of the 110 contacts who had no parent in the health profession, 17 (16%) developed symptoms. This was found to be statistically significant (*p* = 0.01) representing health professionals as index cases being a risk factor for the transmission of COVID-19 to paediatric contacts.

**Figure 3:**
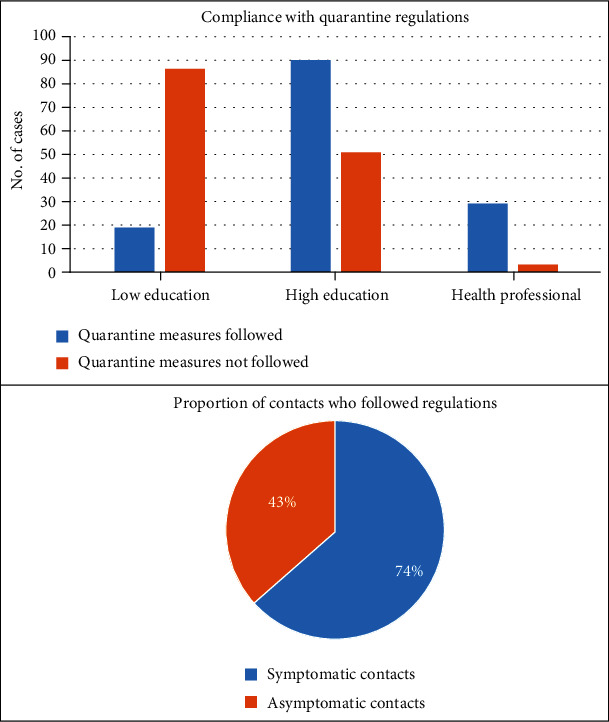
An overview of the compliance with quarantine regulation. Parents of the contacts with higher education and in the health profession and the families of symptomatic contacts reported better compliance with quarantine regulations (*p* ≤ 0.01).

Figure 3 shows an overview of the compliance with quarantine regulation. More than half of the contacts (51%) failed to follow quarantine regulations. Parents of the contacts with higher education and in the health profession and the families of symptomatic contacts reported better compliance with quarantine regulations.

## 4. Discussion

This retrospective study was conducted to assess the spectrum of paediatric contacts of adult COVID-19 patients in Islamabad, Pakistan. This study shows that a significant number of children were exposed to adult patients. This also confirms that the majority of the contacts were asymptomatic or had mild disease. The COVID-testing rate and following of quarantine measures were around 50% in our study.

In our study, the mean age of contacts was 6.6 years. It is similar to the mean age of infected and admitted paediatric patients [10, 11], while a meta-analysis [12] showed a higher mean age of 8.9 years. Our study showed a higher male involvement than females though there was no statistical difference in gender among symptomatic and asymptomatic contacts. This is in accordance with the gender characteristic of affected children by Dong et al. [13]. Each contact had mean exposure of 4.18 ± 0.98 days with a slightly lesser duration of contact in symptomatic contacts though this difference was not statistically significant. In a study from China [14], the duration of contact was 7-8 days while Huang et al. [15] showed that a few hours of contact was also sufficient for the transmission of disease in relatively younger contacts. It shows that contacts of a confirmed case should not be taken lightly and must be observed or traced for symptoms or testing. There was no history of foreign travel or travel to the Sindh province (the most endemic region of the country at that time) in any patient or contacts showing local circulation of the virus in Rawalpindi and Islamabad.

Of the 100 adult COVID-19 patients, there were 137 contacts with 1.3 contacts per patient. This association of contact exposure has not been studied previously. Pakistani under-14 population is around 35.4% [ 16] which is at risk of infection. In this study, 81% of contacts were asymptomatic and 19% were symptomatic. A systematic review presenting data on 2914 paediatric patients with COVID-19 identified that 14.9% of children were asymptomatic [17]. Others have reported 18% asymptomatic cases in a meta-analysis [18] and 16% asymptomatic cases in a European cohort [19]. This lower number of asymptomatic cohorts in these studies is due to the fact that it is from all of the confirmed cases while, in our study, only 56% underwent COVID testing, so the laboratory confirmation in all symptomatic or asymptomatic contacts could not be established. Among symptomatic contacts, fever and cough were the most prevalent symptoms. This is in accordance with other studies regarding symptoms of COVID-19 in children [6, 7, 18]. The disease showed a mild pattern and no admission or death in our study. Same pattern is reflected by the pooled data from seven countries [20].

Younger age and preexisting medical conditions appear to render a higher risk for COVID-19 infections [21]. In our study, some contacts suffered from previous cardiac or respiratory illness or there was history of repeated infections. Out of this, respiratory illness and recurrent infections were statistically significant in terms of symptomatology though we could not identify the exact diagnosis of infections in these cases.

There is a lot of debate about decreased severity and limiting mortality rate due to COVID-19 in countries where BCG vaccination coverage is maximum [22]. It has been observed that the BCG vaccine offers a certain degree of cross-protection against viral illnesses through trained immunity-related mechanisms [23, 24] forming the basis of the observation of reduced disease severity in BCG-vaccinated populations. Several multicenter studies suggest an inverse relationship of decreased COVID-19 morbidity and mortality with higher BCG vaccination status [25–27]. On the other hand, many studies have negated this idea [28, 29]. Our study population was maximally vaccinated for BCG, and a lower number of symptomatic contacts and mild disease favor the protective effect of BCG vaccination in COVID-19 infection. On the other hand, 4 (36%) of the contacts who were given influenza vaccine developed symptoms, but 23 (22%) contacts who did not receive influenza vaccine were symptomatic. However, this difference was not statistically significant. Similar results have been documented by Salem et al. [30] but opposite findings shown by Amato et al. [ 31] leaving us with no conclusive evidence that the influenza vaccine confers any protection against COVID-19.

In our study, health professionals comprised about 10% of the total patients, and 37% of contacts of health professionals developed symptoms as compared to 16% contacts who had no adult source from a health-related setting. This is in accordance with the study by David Koh [32]. To our knowledge, frequency of symptomatic contacts of health professionals has not been studied before.

Iron metabolism and hyperferritenemia are being intensively studied in severe COVID-19 infection. Studies in adults reveal that anemia is a risk factor for severe COVID disease [33]. Keeping this in view, hematinic use was assessed in our paediatric contacts assuming that the children with iron deficiency anemia would be taking it. In our study, more contacts who were taking hematinics were symptomatic as compared to those who were not taking it. This shows an indirect relationship between COVID-19 symptomatology and anemia though we could not establish haemoglobin values due to the lockdown. To our knowledge, this association in the paediatric group has not been previously studied.

Frequency of COVID-19 testing in contacts was 56% in our study. It seems to be quite high as compared to the limited capability of our country in terms of contact tracing and testing [34]. This appears to be due to the small study population having a relatively higher education level and the majority belonging to the upper middle class, thus representing an informed and cautious subgroup.

The strength of the study is that it is a first study looking into different parameters of paediatric contacts. Also, very little is known about paediatric COVID-19 demographics in Pakistan and South Asia prior to it, and this study gives an insight to it. The limitation of our study is that it is a small-scale study, and data was collected over the phone as contacts could not be examined because of the lockdown.

## 5. Conclusion

A significant proportion of children were exposed to adult COVID-19 patients. Cough and fever remained the common symptoms of paediatric contacts as in paediatric patients. Majority of paediatric contacts remained asymptomatic. Children with preexisting medical conditions and with parents in health profession were susceptible to infection. Quarantine measures were not adequately followed in our study. In our region, paediatric contacts of adult COVID-19 patients had the same parameters as paediatric COVID-19 patients from all around the world.

## Figures and Tables

**Table 1:** Characteristics of the adult index cases with COVID-19.

Characteristics	Number (%) of cases		*p* value^1^
Gender			0.001
Male	67		
Female	33		
Socioeconomic status			≤0.001
Poor (<Rs20,000/month)	1		
Lower middle class (Rs20,000-50,000/month)	47		
Upper middle class (Rs50,000-100000/month)	42		
Upper class (>Rs100000/month)	10		
Residence			
Islamabad	65		
Rawalpindi area	21		
Other	14		
Characteristics according to gender			
Outcome	Males	Females	
Discharged from hospital, n (%)	57 (85%)	29 (88%)	ns
Expired, *n* (%)	10 (15%)	4 (12%)	
Number of contacts ∗ number of index cases per contact (total number of contacts)	0∗26 (0)	0∗13 (0)	
	1∗18 (18)	1∗5 (5)	
	2∗15 (30)	2∗4 (8)	
	3∗4 (12)	3∗4 (12)	
	4∗4 (16)	4∗2 (8)	
		5∗4 (20)	
		8∗1 (8)	
Number of contacts per index case	1.13	1.84	0.03

*n*: number; ns: not significant. ^1^Chi-square tests for categorical variables, independent sample *t*-test for numerical variables. The *p* value indicates significant difference with respect to gender and socioeconomic status of the adult index cases. In the lower section, the *p* value indicates difference between male and female index cases with respect to mortality, and number of contacts per index case.

**Table 2:** Characteristics of the paediatric contacts and the risk factors associated with development of symptoms.

	Symptomatic	Asymptomatic	Statistical significance^∗^
	(*n* = 27)	(*n* = 110)	
Males (*n*)	17	58	ns
Females (*n*)	10	52	
Age in years	6.7 (±3.9)	6.6 (±3.5)	ns
Weight in kg, mean (±SD)	16 (±7.8)	22 (±10)	ns
Duration of contact, days, mean (±SD)	4.1 (±0.8)	4.2 (±1)	ns
Fathers' education, *n* (%)			ns
Uneducated	0	1 (1%)	
Primary	2 (7%)	5 (5%)	
High school	7 (26%)	25 (23%)	
College graduate	6 (22%)	46 (42%)	
Masters	3 (11%)	23 (21%)	
Doctor	9 (33%)	10 (9%)	
Mothers' education, *n* (%)			ns
Uneducated	1 (4%)	3 (3%)	
Primary	3 (11%)	21 (19%)	
High school	8 (30%)	25 (23%)	
College graduate	8 (30%)	49 (45%)	
Masters	2 (7%)	4 (4%)	
Doctor	5 (19%)	4 (4%)	
Nurse	0	4 (4%)	
Symptoms, *n* (%)			
Fever	25 (92%)	0	
Cough	18 (67%)	0	
Diarrhea	5 (19%)	0	
Headache	3 (11%)	0	
Flu-like symptoms	3 (11%)	0	
Burning eyes	2 (7%)	0	
Dyspnoea	0	0	
BCG vaccinated, *n* (%)	27 (100%)	104 (95%)	ns
Influenza vaccinated, *n* (%)	4 (15%)	7 (6%)	
History of respiratory illness, *n* (%)	4 (15%)	3 (3%)	0.01
History of cardiac illness, *n* (%)	2 (7%)	2 (2%)	ns
History of recurrent infections, *n* (%)	4 (15%)	2 (2%)	0.015
History of hematinic use, *n* (%)	4 (15%)	5 (5%)	0.002
Test done-public contact tracing, *n*	6	19	ns
Test done privately, *n*	11	41	ns
Positive tests, *n* (public & private)	15 (5 & 10)	6 (0 & 6)	0.002

^∗^Logistic regression with symptoms as dependent variable. *n*: number of cases; SD: standard deviation.

## Data Availability

Underlying data supporting the results of study can be made available on reasonable request.
